# What Recent Announcements Really Mean

**Published:** 1921-07-02

**Authors:** 


### X-RAYS AND CANCER.

### What Recent Announcements Really Mean.

Following their statement to the Press, the West London Hospital gave, last week, a demonstration of a newly acquired *x*-ray apparatus in the treatment of malignant growth. This new mechanism was designed in the Women's Hospital at Erlangen in Bavaria by Dr. Wintz, and truly remarkable results were claimed in connection with uterine and mammary carcinoma.

As was perhaps inevitable, there followed upon the original announcement from the West London a series of general comments in the daily papers, which were, to put it mildly, highly misleading, and one can only regret that a little more caution is not observed among those laymen who speak so glibly about "cures for cancer."

Now this method from Erlangen has certainly cured—or at least clinically cured—a number of cases of early neoplasm, but for *x*-ray treatment to achieve this is indeed nothing new, provided the cancer is accessible and early. *The Times* quotes from an interview with the radiologists at the West London, and has, we are glad to see, explained the matter with considerable clearness.

The point is that this treatment must in no way be considered as a general cure. The Erlangen apparatus is undoubtedly an improvement upon, and a further refinement of, that which preceded it. Apparently its great claim to merit lies in its power to select and concentrate certain portions of the radiation from an *x*-ray tube, and that to a degree of perfection hitherto unattained. It is the same principle of *x*-ray application as in the general sense, but here is a method in which the "dose" given can be far better regulated. In the description quoted by our contemporary, "the fundamental principle of the whole system is that we now know the 'dose' which any cancer requires, and we are in a position to give that 'dose.'" One hesitates a little in accepting "any cancer" because, although to the professional reader the meaning is clear, the erroneous idea of the achievement of a universal cure is at once conceived by the layman.

As a matter of fact, even with this method it is essential that the cancer should be in the earliest stages of its growth. Once the growth of cancer tissue has become extensive—*i.e.*, invaded vital structures or formed secondary deposits in deep-seated organs—there is not only little use in the *x*-ray method, but the medical profession is still—and may always be—practically devoid of a certain curative means. Would that all public comments on this matter had been made with the same reservation displayed by *The Times*. "It must be clearly understood that so far the apparatus or method have not been tested on a great scale in this country, and consequently they cannot be said to have reached the stage where doubt has ceased to exist. The implied suggestion of the hospital authorities that operations should not be performed until their method has been employed should be received with caution. No doubt they have reason for the course they are adopting. At the same time the best advice which can be given to anyone fearing the onset of cancer is to see a good surgeon. We have not yet, it is feared, reached the point at which this advice may be safely disregarded."

